# Food preservative tBHQ modulates Th17/T_reg_ balance through AHR/NRF2 xenosensors and increases the sensitivity of T cells to activation stimuli

**DOI:** 10.1016/j.crtox.2026.100314

**Published:** 2026-07-18

**Authors:** Zoltán Palczert, Krisztina Németh, Nóra Fekete, Éva Pállinger, Márta Békés-Kanalas, Péter Petschner, Edit I. Buzás, Miklós Csala, Viola Tamási

**Affiliations:** a Department of Molecular Biology, Semmelweis University, Tűzoltó u. 37-47, H-1094 Budapest, Hungary; b Department of Genetics, Cell- and Immunobiology, Semmelweis University, Nagyvárad tér 4, H-1089 Budapest, Hungary; c Immunoproteogenomics Extracellular Vesicle Research Group, Hungarian Academy of Sciences, Semmelweis University, Nagyvárad tér 4, H-1089 Budapest, Hungary; d Department of Pharmacodynamics, Semmelweis University, Nagyvárad tér 4, H-1089 Budapest, Hungary; e Center of Pharmacology and Drug Research & Development, Semmelweis University, Budapest, Hungary; f NAP3.0-SE Neuropsychopharmacology Research Group, Hungarian Brain Research Program, Semmelweis University, Budapest, Hungary

**Keywords:** tBHQ, T cells, AHR, NRF2, PPI, Molecular docking

## Abstract

In this study, we investigated the effect of the food preservative tert-butylhydroquinone (tBHQ) on the activation of murine CD4^+^ T cells. We assessed the role of the nuclear factor erythroid 2–related factor 2 (NRF2) and the aryl hydrocarbon receptor (AHR) and conducted protein interaction studies to identify potential molecular targets of tBHQ.

BALB/c mice received tBHQ orally (1.5% [*w*/w]), after which splenic T cells (Th1, Th2, Th17, T_reg_) were analyzed for number and activation sensitivity.

While the number of effector T cells remained unchanged, their response to stimulation was accelerated in tBHQ treated animals. In contrast, T_reg_ cells increased in number after treatment with this food additive but displayed reduction in count upon activation.

*In vitro* experiments showed that tBHQ modulates the differentiation of Th17 and T_reg_ cells. Pretreating these cells with NRF2 inhibitor trigonelline and/or AHR inhibitor TMF (6, 2, 4′-trimetoxi-flavone) altered the effect of tBHQ on these cells.

We found 14 candidate targets of this chemical (AHR, KEAP1, NQO1, RORC, TBX21, IL6, GATA3, BCL2, FOXP3, IFNG, JAK2, ALB, TGFBR1, and HSP90AA1). Network analysis revealed coordinated regulation of pathways associated with inflammation, T cell differentiation, and xenobiotic response.

In conclusion, our studies highlight the importance of the T cell response after tBHQ exposure and suggest multiple proteins and pathways that are relevant to the immunotoxicity of this compound.

## Highlights

• tBHQ elevates the count of Tregs but does not alter the number of effector T cells.
• Effector T cells from tBHQ treated animals have enhanced acceleration to stimuli.
• Number of Tregs from tBHQ treated animals reduces upon activation.
• Differentiation of Th17 and Treg cells is AHR and NRF2 dependent.
• tBHQ has many binding candidates and is a member of various pathways.

## Graphical abstract

**Figure:**
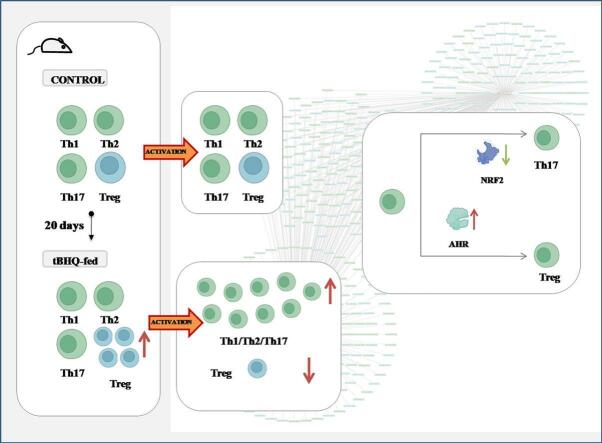

## 1 Introduction

*Tert*-butylhydroquinone (tBHQ) is a common food- and cosmetic additive used as fat antioxidant. It was found to be more effective in many ways than other phenolic antioxidants, *e.g.* butylated hydroxyanisole (BHA) or butylated hydroxytoluene (BHT). Although it is considered to be safe within current regulatory limits, increasing evidence indicates that synthetic phenolic antioxidants may exert dose-dependent biological effects beyond their intended antioxidant function (Xu et al., 2021; Khezerlou et al., 2022; Ren et al., 2025). Although tBHQ is rapidly excreted in the urine (Xu et al., 2021), concerns have been raised regarding its potential carcinogenic and immunotoxic effects (Sherson et al., 2023; Jin et al., 2023; Le Coz and Schneider, 1998; Aalto-Korte, 2000). Human studies disproved carcinogenicity of tBHQ (Zeb, 2020), but immunotoxicity remains poorly characterized.

Emerging experimental evidence suggests that chronic tBHQ exposure modulates immune responsiveness. For instance, tBHQ has been reported to lower the effect of vaccines (Awali et al., 2023; Németh et al., 2022). Moreover, this chemical has been shown to inhibit dendritic maturation and activation (Awali et al., 2026). It also increases the possibility of developing food allergies (Jin et al., 2024) and elevates the secretion of various cytokines (Kensler et al., 2007).

tBHQ is a potent activator of redox-sensitive xenobiotic response pathways, particularly the Kelch-like ECH-associated protein 1 (KEAP1)–NRF2 axis (Xu et al., 2021; Deng et al., 2021). NRF2 regulates antioxidant defense, phase II metabolism, and cellular stress adaptation, but accumulating evidence suggests that NRF2 signaling also shapes inflammatory and immune responses (Audousset et al., 2021; Ryan et al., 2022; Liu et al., 2025).

To date, there is no clear evidence that tBHQ interacts with AHR to induce T cell differentiation or that tBHQ induced AHR plays a functional role in immune processes.

AHR mediated signaling alters the differentiation of T_reg_ and Th17 cells, and the effect depends on the type of the ligand and the strength of the signal (Bahman et al., 2024; Sznurkowska, 2025). Numerous xenobiotics, as well as microbiome-derived or dietary compounds have been shown to have an impact on the immune responses through AHR (Coumoul et al., 2026; Yongkai et al., 2024). Since there is a functional crosstalk between AHR and NRF2, and NRF2 can be activated with tBHQ, it seems plausible this chemical induces a coordinated regulation of detoxification and immune processes through these two receptors (Deng et al., 2021). The crosstalk between AHR and NRF2 prompted us to investigate the effect of tBHQ on AHR, in addition to NRF2. While the immunological role of AHR has been extensively investigated in various tissues and immune diseases, the contribution of NRF2 to T cell biology remains less clearly defined. Experimental studies suggest that NRF2 induction can alter T cell activation, differentiation, and metabolic programming (Ryan et al., 2022; Johnson et al., 2010; Cuadrado et al., 2019; Tripathi et al., 2025), yet the mechanistic basis and *in vivo* relevance of these effects are not fully understood.

Because humans are continuously exposed to tBHQ through food and consumer products, repeated activation of redox-sensitive signaling pathways may influence immune homeostasis. However, exposure is often intermittent. Therefore, subchronic dietary dosing is a good model of occasional exposures to this compound. Whether it is sufficient to alter immune responses remains unclear and the potential involvement of the NRF2 and AHR signaling pathways in these processes is still poorly understood. This knowledge gap prompted us to investigate how subchronic oral tBHQ exposure affects T cell activation sensitivity and differentiation, with particular focus on AHR–NRF2-associated signaling pathways.

From a mechanistic toxicology perspective, xenobiotic-induced redox changes can act as a molecular initiating event (MIE) that ultimately lowers T cell activation thresholds and influences their differentiation. Perturbation of AHR- and NRF2-dependent transcriptional programs may represent early key events influencing the balance between effector (Th1, Th2, Th17) and regulatory (T_reg_) responses. Clarifying whether dietary exposure shifts activation sensitivity, differentiation potential, or immune homeostasis is therefore relevant to immunotoxic risk assessment.

Based on these considerations, we hypothesized that subchronic, oral exposure to tBHQ changes the CD4^+^ T cell mediated responses through altered activation sensitivity and modulation of AHR- and NRF2-dependent signaling of Th17/T_reg_ cells. To test this hypothesis, we combined *in vivo* dietary exposure in BALB/c mice with *ex vivo* functional activation assays, pharmacological pathway inhibition during differentiation, and integrative *in silico* target prediction. This approach was designed to identify mechanistic links between xenobiotic receptor activation and downstream immune functional outcomes relevant to immunotoxicity.

Here, we report that a prolonged tBHQ containing diet (20 days) increases the percentage of regulatory T cells (T_reg_) among lymphocytes in mice. However, it also increases the sensitivity of effector T cells to activating stimuli by reducing the reaction time to such activators and reduced the count of T_reg_ cells. This collectively suggests a type of immune response, where increased tolerance is associated with higher sensitivity of effector T cells. We also show that Th17 and T_reg_ differentiation after tBHQ exposure involves the xenosensors AHR and NRF2. As tBHQ modulates many immunological pathways, we conducted binding studies to identify potential tBHQ-interacting proteins implicated in T cell differentiation.

## 2 Materials and methods

### 2.1 Animals

The *in vivo* experiments aimed to investigate the effect of tBHQ on immune cells in the spleen. The use of mice, specifically the BALB/c strain, was chosen due to their well-characterized immune system and widespread use in immunological studies. Only adult males were included to reduce variability related to hormonal cycles; however, the exclusion of females should be noted as a limitation that may affect our findings. Male BALB/c mice (wild type, age between 12 and 13 weeks, 20-30 g) were obtained from a certified supplier and maintained as specific-pathogen free (SPF). Mice were housed under a normal light-cycle (12 h light - 12 h dark). The animals were provided with water and food *ad libitum.* The animals were fed a tBHQ-supplemented (1.5% *w*/w; Fluka, USA) or a control diet for 20 days. Body weight of mice was monitored regularly (Supplementary Fig. 1). Food pellets were prepared as follows: AIN 76-A semipurified diet (MP Biomedicals, Solon, OH, USA) was mixed with 1.5% tBHQ (*w*/w), 8% warm distilled water (w/w), and 3% gelatine (w/w). After cooling, the mixture was shaped into food pellets. Control diet (vehicle) was prepared the same way, without tBHQ. Higher dietary dose (1.5% w/w tBHQ) was applied to allow clear detection in immunomodulatory effects of tBHQ *in vivo* (the dose is comparable to that used in analogous animal models, *e.g.* 1% (*w*/w) tBHQ, even for longer exposure times than 20 days) (Khezerlou et al., 2022; Hu et al., 2017; Liu et al., 2016). In our experiments, the animals did not show signs of toxicity. After the treatment period, mice (6 animals/group) were sacrificed using CO2 asphyxiation. All animal experiments followed the European Community Council Directive of 24. Animal welfare was continuously monitored, and any signs of distress or pain were managed according to institutional guidelines. All procedures complied with the European Community Council Directive of 24 November 1986 (86/609/EEC) and were approved by the Institutional Animal Care and Use Committee.

### 2.2 *Ex vivo* cell characterization experiments

**Fig. 1:**
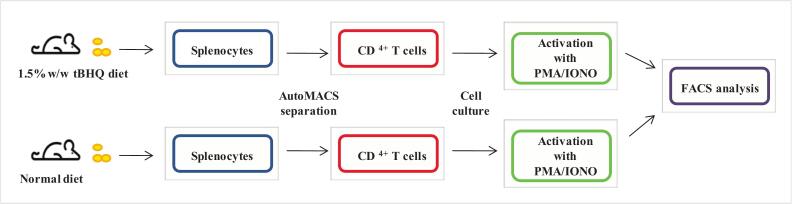
Experimental setup of *ex vivo* cell characterization. Mice were fed with tBHQ for 20 days and after sacrification, splenic CD4+ cells were separated with autoMACS separator. After that, cells were activated with PMA/ionomycin and count of various T cells was analyzed with flow cytometry.

Splenic CD4^+^ cells were isolated from *per os* tBHQ-treated and control mice with magnetic separation using AutoMACS separator (Miltenyi Biotec, Germany) according to the instructions of the manufacturer. Lymphocytes that were > 85% pure CD3^+^CD4^+^ cells were used in downstream experiments. (Supplementary Fig. 1). After isolation, cells were treated with brefeldin A (10μg/ml, 3 h, Sigma, USA). One half of cells from each animal group were subject to short (3 h) activation (25 ng/ml *phorbol myristate acetate [PMA] and* 1 μg/ml ionomycin [IONO]), the other half was further processed without activation. The used activation time was shorter than what is recommended for cytokine production, since the cells were already exposed to tBHQ *in vivo* prior to *ex vivo* PMA/IONO restimulation. Therefore, the isolated T cells were not naive cells undergoing primary activation *in vitro*, but rather cells previously exposed to systemic immunomodulatory effects *in vivo*. Under these conditions, we aimed to capture early recall/activation responses while minimizing prolonged pharmacological overstimulation induced by PMA/IONO (Mandala et al., 2021). T cell subsets were analyzed by flow cytometry (FACS Calibur, Beckton Dickinson, USA) (Fig. 1).

### 2.3 T cell differentiation

Isolated CD4^*+*^ cells were differentiated with specific differentiation kits in anti-CD3 coated activation plates (3 plates/group) either into regulatory T cells (T_reg_) (FlowCellect™ Mouse T_reg_ Differentiation Tool Kit, Merck, Millipore Germany) or Th17 cells (FlowCellect™ Mouse Th17 Differentiation Tool Kit, Merck, Millipore, Germany) in the presence/absence of 10 μM tBHQ, a specific AHR inhibitor TMF (6, 2, 4′-trimetoxi-flavone, 1 μM; Sigma, USA) or a specific NRF2 inhibitor trigonelline (TRIG) (1 μM; Sigma, USA), respectively. TMF and tBHQ were dissolved in DMSO and subsequently diluted in cell culture media. TRIG was dissolved in PBS and further diluted in cell culture media. In case of each sample, the final concentration of DMSO was adjusted to 0.1%. Importantly, we found that such a small concentration of DMSO is not toxic to the cells (data not shown). Vehicle-treated cells (0.1% DMSO) served as control groups. Cultured naive CD4^+^ cells were pre-treated with the mentioned, properly diluted AHR and/or NRF2 inhibitors for 1 h before addition of tBHQ. After that, reagents for Th17/T_reg_ differentiation were added according to the manufacturer's protocol, and the differentiation lasted 6 or 7 days for Th17 or T_reg_ cells, respectively on CD3 coated activation plates. Treatments were repeated after each media change. T cells were submitted to FACS analysis, and data were presented as percentage of CD4^+^ lymphocyte-enriched fractions.

### 2.4 Flow cytometry, antibodies

Isolated lymphocytes were stained with fluorochrome-conjugated antibodies against surface molecules. These included anti-CD3 (2 μl CD3-PE, anti-mouse, BD Biosciences, USA), anti-CD4 (5 μl CD4-PerCP, anti-mouse, Merck Millipore, USA, 5 μl CD4-PerCP/Cy5.5, anti-mouse, Merck Millipore, USA or 5 μl anti-CD4-FITC, Miltenyi Biotec, Germany), and anti-CD25 (2 μl CD25-PE, anti-mouse, Merck Millipore), and/or intracellular T-cell specific proteins, such as anti-IFNγ (2 μl IFNγ-PE, anti-mouse, Sony, USA), anti-IL4 (1 μg IL4-PerCP-Cy5.5, anti-mouse, BD Biosciences, USA), anti-IL-17 ([5 μl IL17-FITC, Merck Millipore), and anti-Foxp3 (2 μl Foxp3-Alexa Fluor488 antimouse Merck Millipore). Viability of the lymphocytes were analyzed with Fixable Viability Dye (eFluor660, Merck Millipore).

The antiCD25-PE and antiFoxp3-Alexa Fluor488 antibodies were included in the FlowCellect™ Mouse Viable T_reg_ characterization kit (Merck Millipore, Germany). Anti-CD17-FITC and anti-CD4-PerCP were part of FlowCellect™ Mouse Th1/Th17 Intracellular Cytokine kit (Merck Millipore, Germany).

All stained cells were analyzed with FACS Calibur flow cytometer (Becton Dickinson, Fullerton, CA), and the results were interpreted with on-line platform Floreada.io (https://floreada.io/ (accessed in November 2023; the last update was carried out in June 2023)). For all gating strategies see Supplementary Fig. 2.

### 2.5 Target collection and molecular docking prediction

SwissTargetPrediction (Daina et al., 2019), PubChem (Kim et al., 2025), and PharmMapper (Wang et al., 2017) were utilized to identify the target collection for tBHQ. 14 proteins associated with T cell differentiation were selected based on the binding energy. The crystal structures of the proteins were obtained from the Protein Data Bank (PDB), while the 3D structure of tBHQ was retrieved from the PubChem database. The core proteins were processed by Pymol to remove water molecules and original ligands. Then, CB-DOCK2 was applied for molecular docking and prediction of the binding free energy (Liu et al., 2022a). For each case, five docking conformations were generated, and the conformation with the lowest binding free energy was selected for prediction.

### 2.6 Protein-protein interaction network

Protein networks were constructed for each of the 14 proteins predicted to be tBHQ targets individually, using the STRING database (Szklarczyk et al., 2017). The organism was set to *Homo sapiens*, and a confidence level greater than 0.4 was used to identify genes with reliable interactions. These genes were then analyzed to determine the active proteins corresponding to the target genes. The program was also configured to display a maximum of 10 potential interactions. The results were imported into Cytoscape software (Kohl et al., 2011) and merged to present a PPI network including our 14 target proteins. Cytoscape was developed for the visualization and analysis of biological processes through networks. It is designed to calculate the topological characteristics of the input nodes and edges, and to generate a protein–protein interaction (PPI) network diagram using the node degree as a standard. In a PPI analysis, the degree value indicates the number of connections between a specific gene and other genes within the network. A higher degree value signifies a stronger connectivity of the gene within the network, suggesting that it may play a more important role in the biological process examined. Concurrently, the Cytohubba plug-in was utilized to cluster the input proteins from this topology, thereby facilitating the identification of potential critical genes or proteins by calculating the importance of the nodes in the network (Chin et al., 2014). To this end, a cluster analysis was performed to examine six aspects: maximum clique centrality (MCC), maximum neighborhood centrality (MNC), degree, eigenvector centrality (EC), closeness, and betweenness. These aspects were then used to estimate the importance, centrality, and criticality of the proteins. The objective of this analysis was to identify the core action targets. For validation, GO/KEGG functional annotation clustering was used. DAVID Bioinformatics was utilized to perform Gene Ontology (GO) and Kyoto Encyclopedia of Genes and Genomes (KEGG) functional annotation clustering, with the objective of validating the association between the genes in question and T cell differentiation (Table 1, Supplementary Table 1). In addition, G:Profiler was used to present additional, alternative pathways and biological relationships in which these genes are also involved (Supplementary Table 2).

**Table 1:** GO and KEGG functional annotation clustering by DAVID Bioinformatics. Categories, representative terms, associated p-values, and genes are shown. (Enrichment treshold: 0.05).

Name	p-value	Genes
**Cell developement**		
hsa04659:Th17 cell differentiation	2.591447954207353E-13	IL6, IFNG, TBX21, RORC, GATA3, AHR, JAK2, FOXP3, TGFBR1
GO:0008284 ∼ positive regulation of cell population proliferation	1.9746431222178063E-4	IL6, IFNG, BCL2, JAK2, TGFBR1
hsa04630:JAK-STAT signaling pathway	0.001446584353117216	IL6, IFNG, BCL2, JAK2

**Diseases**		
hsa05321:Inflammatory bowel disease	1.80165335165096E-8	IL6, IFNG, TBX21, RORC, GATA3, FOXP3
GO:0009615 ∼ response to virus	3.920705385181122E-5	IFNG, TBX21, GATA3, FOXP3
hsa05200:Pathways in cancer	3.856550278658965E-5	NQO1, IL6, IFNG, BCL2, KEAP1, JAK2, TGFBR1
hsa04933:AGE-RAGE signaling pathway in diabetic complications	3.2758125787533065E-4	IL6, BCL2, JAK2, TGFBR1
hsa05161:Hepatitis B	0.0013257702800118375	IL6, BCL2, JAK2, TGFBR1
hsa05152:Tuberculosis	0.0018213232533246541	IL6, IFNG, BCL2, JAK2
hsa05168:Herpes simplex virus 1 infection	0.0018213232533246541	IL6, IFNG, BCL2, JAK2

**Genomics**		
GO:0045893 ∼ positive regulation of DNA-templated transcription	1.8172373405621174E-6	IL6, TBX21, RORC, GATA3, AHR, FOXP3, TGFBR1
GO:0045944 ∼ positive regulation of transcription by RNA polymerase II	3.7014075449218327E-5	IL6, IFNG, TBX21, GATA3, AHR, JAK2, FOXP3
GO:0000122 ∼ negative regulation of transcription by RNA polymerase II	1.6880780326518835E-4	IFNG, TBX21, RORC, KEAP1, GATA3, FOXP3
GO:0045892 ∼ negative regulation of DNA-templated transcription	3.038978743244564E-4	IFNG, TBX21, GATA3, AHR, FOXP3
GO:1990837 ∼ sequence-specific double-stranded DNA binding	3.0719663291672844E-4	TBX21, RORC, GATA3, AHR, FOXP3
GO:0003700 ∼ DNA-binding transcription factor activity	4.0317157635961305E-4	TBX21, RORC, GATA3, AHR, FOXP3
GO:0001227 ∼ DNA-binding transcription repressor activity, RNA polymerase II-specific	5.582477471730564E-4	TBX21, RORC, GATA3, FOXP3
GO:0000785 ∼ chromatin	0.0030496260862183577	TBX21, RORC, GATA3, AHR, FOXP3
GO:0000981 ∼ DNA-binding transcription factor activity, RNA polymerase II-specific	0.0058745294141108505	TBX21, RORC, GATA3, AHR, FOXP3
GO:0003677 ∼ DNA binding	0.010929592043963559	ALB, RORC, GATA3, AHR, FOXP3
GO:0000978 ∼ RNA polymerase II cis-regulatory region sequence-specific DNA binding	0.03301300056340416	TBX21, RORC, GATA3, FOXP3

### 2.7 Statistical analysis

**Fig. 2:**
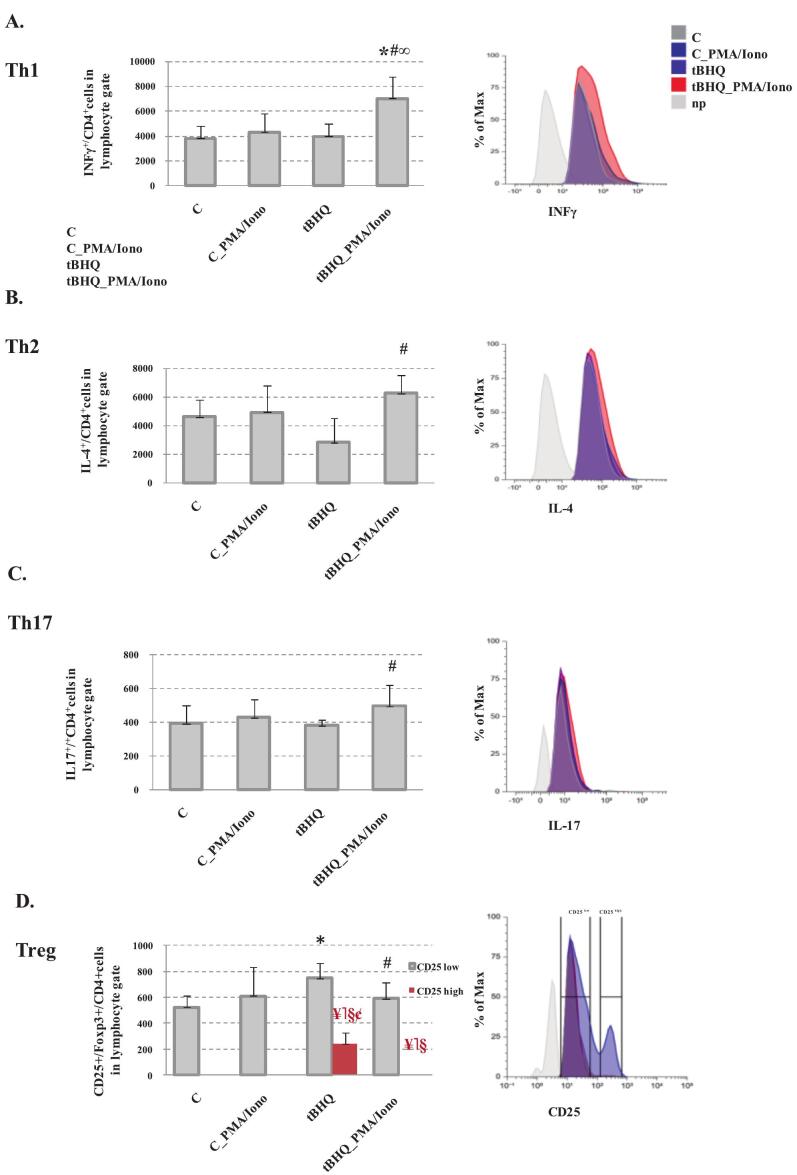
Effect of tBHQ containing diet and activation by PMA/Iono on Th1, Th2, Th17 and Treg cell counts. Splenic CD4+ cells were isolated from tBHQ treated or control BALB/c mice and divided into activated (3 h with PMA/ionomycin) and not activated groups. Th1 (A), Th2 (B), Th17 (C) or Treg (D) cells were counted by FACS analysis in CD4+ lymphocyte gate. Representative histograms are shown on the right for each group (C_control: Not activated after control diet; C_PMA/Iono: Activated after control diet; tBHQ: not activated after tBHQ-containing diet; tBHQ_PMA/Iono: Activated after tBHQ-containing diet; np: Negative population). Statistical significance for Th1/2/17, and CD25low Treg (gray column) compared to C: *p < 0.05; to tBHQ: #p < 0.05; to C_PMA/Iono: ∞*p* < 0.05. Statistical significance for CD25high Treg tBHQ (red column): Compared to C: ¥p < 0.05; to C_PMA/Iono: ˥p < 0.05; to tBHQ_PMA/Iono: §p < 0.05; to Treg tBHQ_PMA/Iono: ¢p < 0.05. (*n* = 6 mice/group). For statistical analysis, two-tailed student's *t*-test was used. For gating strategy, see Supplementary Fig. 1, 2 and 3. (for interpretation of the references to color in this figure legend, the reader is referred to the web version of this article.)

**Fig. 3:**
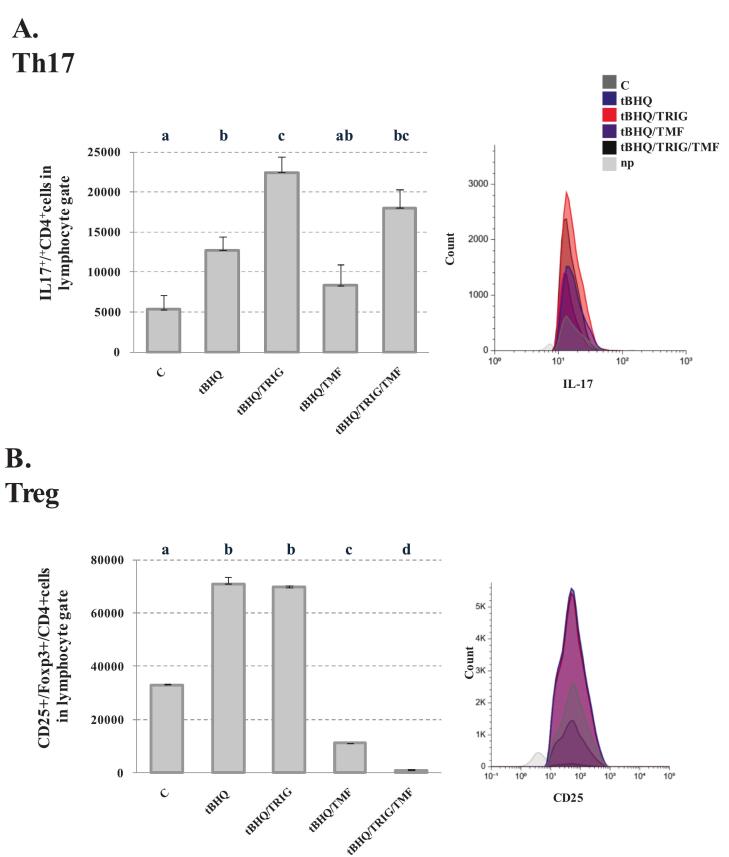
Involvement of AHR and NRF2 receptors in Th17 and Treg differentiation upon tBHQ treatment. Splenic CD4+ cells were differentiated on anti-CD3 coated activation plates either into Th17 (A) or Treg (B) cells in the presence or absence of tBHQ (10 μM), AHR inhibitor TMF (1 μM) or NRF2 inhibitor TRIG (1 μM) as indicated. Cells treated with 0.1% DMSO were used as control (C). Th17 or Treg cell number was determined with FACS analysis. Representative histograms are shown for each group on the right; np: Negative population). Statistical significance was determined by one-way ANOVA followed by Tukey's multiple comparisons test. Groups not sharing a common letter (a, b, c, d) differ significantly (p < 0.05). Data are presented as mean ± SD (*n* = 3). For gating strategy, see Supplementary Figs. 4 and 5.

Beside specific statistical methods used for *in silico* analysis, the results were expressed as mean ± standard deviation (SD). In case of Fig. 2, the statistical significance between two groups was determined using a two-tailed Student's *t*-test. For the data presented on Fig. 3, comparisons among multiple groups were performed using one-way analysis of variance (ANOVA) followed by Tukey's *post hoc* multiple comparisons test. *P* values <0.05 were considered statistically significant.

## 3 Results

### 3.1 Effect of tBHQ treatment on the activation sensitivity of CD4^+^ T cells

To investigate whether tBHQ modulates T cell differentiation, flow cytometry analysis was performed (see experimental setup on Fig. 1). Splenic CD4^+^ T cells isolated from tBHQ-treated and control animals were studied with or without activation by PMA/Ionomycin. Control not activated (C), and tBHQ-treated not activated (tBHQ), as well as control activated (C_PMA/Iono), and tBHQ-treated activated (tBHQ_PMA/Iono) T cells were analyzed by flow cytometry, and the percentages of CD4^+^ lymphocyte fractions were compared in the four groups (Fig. 2).

The number of IFNγ^+^CD4^+^ cells (Th1) was significantly increased in tBHQ_PMA/Iono cells compared to tBHQ (*p* < 0.05, Fig. 2A) and other groups (*p* < 0.01). Parallel experiments with control animals showed no change in the number of Th1 cells after the short activation period we applied.

The number of IL-4^+^CD4^+^ cells (Th2) was significantly higher in the tBHQ_PMA/Iono group compared to tBHQ cells (p < 0.01) (Fig. 2B). Th2 cell counts in the C_PMA/Iono did not differ from those in the C group, neither did tBHQ treatment change the initial/homeostatic number of Th2 cells compared to the control (tBHQ *vs* C).

Similarly to Th1 and Th2 cells, the percentage of IL17^+^CD4^+^ cells (Th17) among CD4^+^ lymphocytes was also significantly higher in the tBHQ_PMA/Iono group compared to the tBHQ group (*p* < 0.05, Fig. 2C). No significant change was measured in the control upon activation, *i.e.*, C_PMA/Iono compared to C.

Functional T_reg_ cells are normally characterized by the expression of the transcription factor FOXP3 (Forkhead box P3) and show a CD4^+^CD25^high^ phenotype. CD25^low^FOXP3^+^ cells are considered to be less stable and partially suppressive (Sznurkowska, 2025). In our experiments, CD4^+^CD25^high^ cells showed a significantly increased number only after tBHQ treatment (*p* < 0.001, Fig. 2D, red). After activation of tBHQ treated CD4^+^ lymphocytes, the count of CD4^+^CD25^high^ fraction was more than 50 times lower (p < 0.001, Fig. 2D), and a slight but significant decline was also observed in CD25^low^ population (*p* < 0.05). T_reg_ in the two control groups did not respond to PMA/Iono activation in such a short time.

### 3.2 Role of NRF2 and AHR xenosensors in the effect of tBHQ on T cell differentiation toward Th17 and T_reg_

In addition to studying the effects of tBHQ on *ex vivo* T cell activation, we were also interested in whether the two xenosensors NRF2 and AHR are involved in the regulation of T_reg_ and Th17 cell differentiation (Fig. 3).

Our results show that tBHQ significantly increased the proportion of Th17 cells among lymphocytes p < 0.05). This effect of tBHQ was largely amplified by co-treatment with NRF2 inhibitor TRIG (*p* < 0.0.005). Marginally significant elevation in Th17 cell count was found in the presence of both inhibitors compared to tBHQ (*p* < 0.07). Administration of TMF alone reduced the proportion of these cells compared to tBHQ alone, however it was not significant (Fig. 3A).

In similar experimental setup, naive splenic T cells were differentiated specifically toward T_reg_, and the proportion of T_reg_ cells was analyzed with FACS. tBHQ treatment significantly increased the number of T_reg_ cells (*p* < 0.001), and this effect was completely counteracted by the AHR inhibitor TMF (*p* < 0.0001). Interestingly, coadministration of TRIG did not affect the tBHQ-boosted number of T_reg_ cells, while further enhanced the effect of TMF, thus the two inhibitors together resulted in a very small proportion of T_reg_ cells among lymphocytes compared to either control (p < 0.0001) or tBHQ-treated cells (p < 0.0001) (Fig. 3B).

### 3.3 Predictions of tBHQ-protein binding

**Fig. 4:**
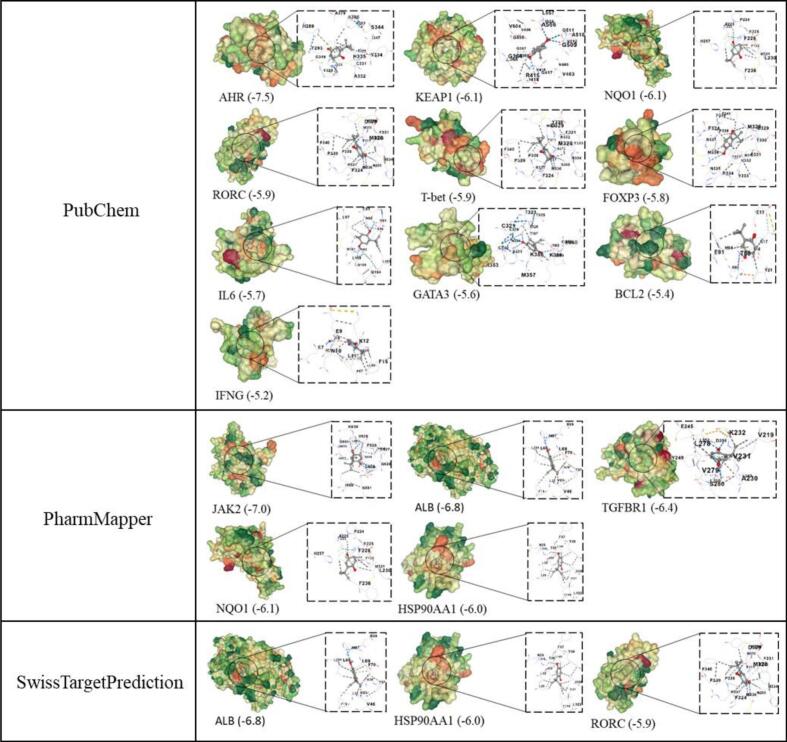
Molecular docking of tBHQ to the target proteins. The on line tools revealing the target proteins are indicated in the left column. The numbers following the identifiers show the binding energy of each protein to the tBHQ molecule. All values are between −5.2 kcal/Mol and − 7.5 kcal/Mol, according to moderate or strong affinities. The colors represent the level of hydrophobicity: The most hydrophobic parts are marked in red. (For interpretation of the references to color in this figure legend, the reader is referred to the web version of this article.)

Potential protein targets of tBHQ have been sought using *in silico* prediction tools. 10 potential targets were identified using PubChem: AHR, KEAP1, NQO1, RORC, T-b et, FOXP3, IL6, GATA3, BCL2 and IFNG. NQO1 and 4 additional targets: JAK2, ALB, TGFBR1, HSP90AA1 were predicted by PharmMapper. SwissTargetPrediction was also able to find 3 of the above-mentioned proteins: ALB, HSP90AA1, and RORC. Molecular docking was performed with CB-Dock2, and it confirmed potential tBHQ binding to these target proteins (Fig. 4). Affinity was considered as very weak or missing when the estimated binding energy was above −5 kcal/mol. Binding energies between −7 and − 5 kcal/mol were regarded as moderate binding capacity; and binding energies equal to or below −7 kcal/mol constituted the criterion for high affinity and strong binding capacity. We found strong binding capacity for AHR and JAK2, while based on binding energy values, ALB, IL6, KEAP1, NQO1, RORC, T-bet, FOXP3, GATA3, BCL-2, IFNG, TGFBR1 and HSP90AA1 bind to tBHQ with moderate strength.

### 3.4 Protein-protein interaction network

**Fig. 5:**
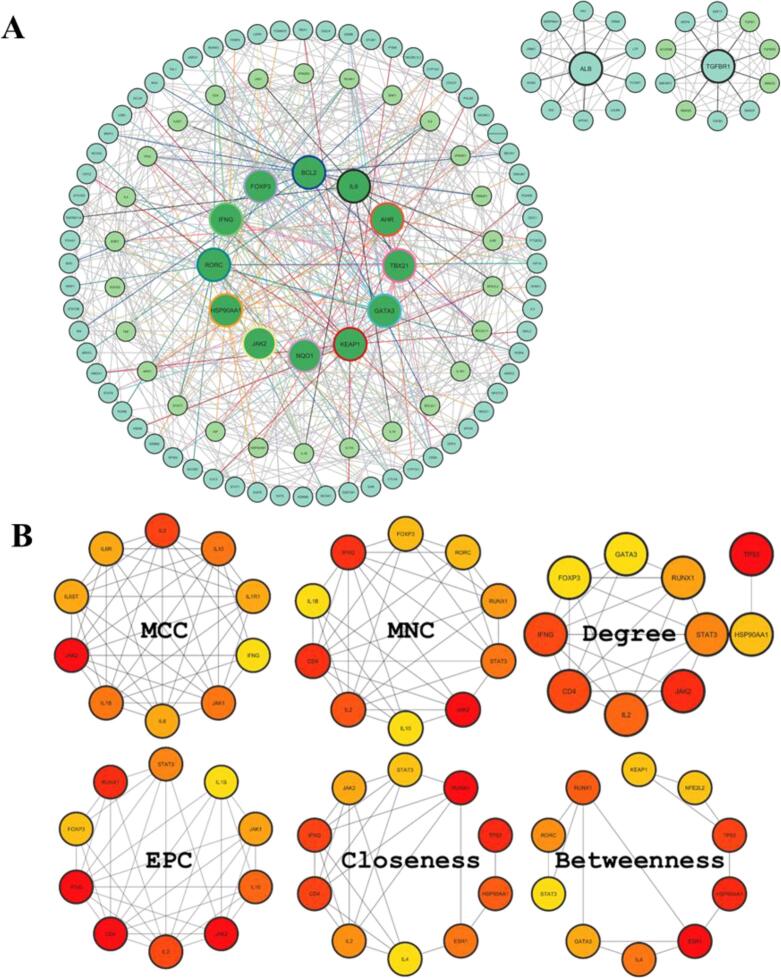
Protein-protein interactions and Cytohubba analyses of the core target proteins. (A) The large nodes represent the core target proteins, while the small nodes indicate the proteins that interact with them. A degree filter was applied to highlight nodes that have at least ten in/out edges showing their importance. These nodes are marked with light green. The edges connecting each small node to the core targets are marked with unique colors, and the edges connecting small nodes are gray. (B) Top hub gene networks by incorporating 6 algorithms: maximal clique centrality (MCC), maximum neighborhood centrality (MNC), degree of nodes, edge percolated component (EPC), closeness, and betweenness. The proteins with the highest values are considered the most important hub genes and are displayed in dark red. In contrast, proteins with lower values are considered less significant and are marked with a light-yellow color, so the color shade indicates the ranking of each protein in the network. (for interpretation of the references to color in this figure legend, the reader is referred to the web version of this article.)

To determine relevant protein interactions of the 14 predicted tBHQ targets, protein-protein interaction network analysis was performed. Network data were imported from STRING database, and visualization was performed by Cytoscape (Fig. 5A). Further clustering was completed using CytoHubba analysis, which revealed the importance of our targets from different perspectives, including maximum clique centrality (MCC), maximum neighborhood centrality (MNC), degree, eigenvector centrality (EC), closeness, and betweenness (Fig. 5B). We validated the networks using GO/KEGG analysis and a selection of most significant terms is represented in Table 1 and Supplementary Table 1. Pathways with significant enrichment (*p* < 0.05) were clustered into three main categories: “Cell development”, “Diseases”, and “Genomics”. Within the cell development category, Th17 cell differentiation (GO: 0046651) and positive regulation of cell population proliferation were among the most significantly enriched terms. Disease-related clusters included pathways such as JAK-STAT signaling, inflammatory bowel disease, and viral infections. Genomic clusters were dominated by transcriptional regulation processes, including RNA polymerase II – specific DNA-binding transcription factor activity. Several key genes, such as **IL6, IFNG, TBX21, RORC** and **FOXP3**, were represented across multiple clusters, indicating their potential roles in multiple biological processes. Categories, representative terms, associated *p*-values, and genes are shown in Table 1.

## 4 Discussion

In this study, we show that the widely used food additive tBHQ increases the proportion of regulatory T cells (T_reg_) after 20 days of oral treatment, while the proportions of effector T cell subsets (Th1, Th2, and Th17) remain unchanged. However, *ex vivo* stimulation with PMA/ionomycin revealed that effector T cells from tBHQ-treated mice respond more rapidly to activating stimuli. Under the same stimulation conditions, the proportion of T_reg_ cells decreased *ex vivo*. Furthermore, our results indicate that tBHQ influences the differentiation of Th17 and T_reg_ cells. Although the effects of tBHQ on Th1/Th2 cells have been thoroughly investigated in rodents (Rockwell et al., 2012) and *in vitro* models (Zagorski et al., 2013), very few studies have examined its impact on splenic Th17 and T_reg_ cells in *ex vivo* mouse models and on the differentiation of these cells from naive CD4+ T cells. Therefore, in our differentiation-related experiments, we focused primarily on these cell types and extended our investigation to the role of AHR and NRF2 transcription factors in their differentiation. Our findings indicate that AHR and NRF2 may participate in the regulation of Th17 and T_reg_ differentiation, although the exact contribution of these pathways remains uncertain and needs further studies. Finally, we sought proteins with potential binding sites for tBHQ presumably modulating AHR and/or NRF2 expression.

Studies show that T cells are central players in tBHQ mediated immune response (Turley et al., 2015; Freeborn et al., 2019; Boss et al., 2018). As our experiments reveal, tBHQ increases the activation sensitivity of effector T cells and thus promotes a switch to an autoreactive phenotype (Fig. 2). One explanation for the results of *ex vivo* studies is that tBHQ regulates intracellular Ca^2+^ levels by blocking the sarco/endoplasmic reticulum Ca^2+^-ATPase (SERCA) in the membrane of the endoplasmic reticulum. Ca^2+^ oscillations are critical in T cell activation, as natural activation of these cells also depends on Ca^2+^ influx triggered by antigen mediated stimulation of the T cell receptor (TCR) (Gallagher et al., 2021; Jakobs et al., 2024; Uddin and Thomas, 2025). In cell culture, Ca^2+^ release can be induced with a PMA/IONO mixture. Notably, tBHQ-induced Ca^2+^ changes only partially activate the same pathways as TCR-mediated antigen presentation, and the underlying early mechanisms remain to be explored.

Although PMA/ionomycin bypasses proximal TCR signaling, enhanced responsiveness in tBHQ-exposed cells suggests that downstream signaling thresholds are shifted. We also observed increased LC3A-II levels (data not shown), indicating enhanced autophagy, a Ca^2+^-linked process implicated in CD4^+^ T cell activation and differentiation (Jeong et al., 2022). Together, these findings suggest that tBHQ may reprogram activation sensitivity *via* redox/Ca^2+^/autophagy crosstalk.

Earlier studies have shown that xenosensors such as AHR and NRF2 influence the regulation of immune responses (Rockwell et al., 2012; Schiering et al., 2017). Given that tBHQ is a known activator of NRF2 (Khezerlou et al., 2022; Liu et al., 2025; Li et al., 2011), we examined the role of both receptors in Th17/T_reg_ differentiation. We used pharmacological inhibition of NRF2 and AHR with TRIG and TMF, respectively.

It should be noted that TRIG- and TMF-only treatment groups were not included in these studies, since the objective of the pharmacological inhibition was to investigate the contribution of NRF2 and AHR signaling to the effects of tBHQ rather than the independent biological effects of the inhibitors. Therefore, the off-target effects of TRIG and TMF, whether administered alone or in combination cannot be excluded, which should be considered as limitations of the present study. Our findings indicate a potential promoting role of AHR and inhibitor function of NRF2 in Th17 cell differentiation following tBHQ exposure. tBHQ-induced T_reg_ differentiation appeared to be more dependent on AHR-related pathways, as it was substantially attenuated by AHR inhibition, whereas NRF2 inhibition alone exerted only minor effects. The stronger reduction of T_reg_ cells observed upon combined inhibition may suggest a functional interplay between these two xenosensors. Since both receptors are involved not only in T-cell differentiation but also in the regulation of oxidative stress responses (*e.g.*, through activation of *Nqo1*, *Cyp1a1* or *Gsta2* gene), this possibility should also be considered (Grishanova and Perepechaeva, 2022; Huchzermeier and van der Vorst, 2025). Therefore, the observed effects may reflect alterations in cellular stress responses or general reduction in differentiation capacity, and not necessarily pathway specific synergy between AHR and NRF2.

Nevertheless, these findings are consistent with previous results showing that AHR and NRF2 signaling influence each other and can affect T cell polarization under different conditions (Bahman et al., 2024; Tripathi et al., 2025; Rothhammer and Quintana, 2019). Regulation of T_reg_ differentiation by AHR appears to be cell type and concentration dependent. For example, while TCDD induces T_reg_ differentiation, the differentiation of T_reg_ cells is reduced and the differentiation of Th17 cells is increased in the presence of FICZ (Marshall and Kerkvliet, 2010; Li et al., 2025). Downstream mechanisms of NRF2 activation are also dependent on the nature of the triggering chemical. Similarly, NRF2 activation has ligand-specific effects, with some xenobiotics like EGCG promoting T_reg_ and autoimmunity protection, while others have opposite effects (Du and Mohan, 2016; Silva-Islas and Maldonado, 2018). Therefore, it would be important to investigate the influence of each chemical compound on immune cells individually. Persistent activation of the AHR pathway under chronic oxidative stress conditions may contribute to immune dysregulation and tissue injury. The known crosstalk between oxidative stress pathways, (*e.g.*, NRF2 activation) and AHR-associated signaling, suggests that prolonged tBHQ exposure may influence immune homeostasis and inflammatory responses indirectly through AHR (Edamitsu et al., 2022). Therefore, we acknowledge that additional mechanistic studies are required to demonstrate the effect of tBHQ on AHR/NRF2 activation, *e.g.*, with canonical downstream target induction (CYP1A1, CYP1B1).

In this study, we also predicted proteins that have potential binding sites for tBHQ. The candidates were selected using 3 databases, and their corresponding crystal structures were imported from PDB. Proteins with binding energies below −5 kcal/mol, *i.e.*, with moderate or strong affinities are listed on Fig. 4. Using PharmMapper, we identified several proteins involved in immune responses. Our *in silico* analyses identified 14 potential protein targets of tBHQ. As expected, KEAP1 showed strong predicted binding, supporting canonical NRF2 activation. Interestingly, AHR was also identified among potential targets. Although no *in vitro* or *in vivo* evidence currently supports direct binding between AHR and tBHQ, predictive studies suggest that tBHQ may interact with AHR with lower affinity than classical AHR ligands (Mosavi et al., 2024). To demonstrate classical AHR activation, transcriptomic analysis is necessary for specific AHR target genes, such as CYP1A1, CYP1B1, AHRR or TiPARP (Torti et al., 2021).

Notably, JAK2 was also predicted to bind tBHQ with high affinity, providing a possible mechanistic link to cytokine-driven T cell differentiation (El Houdi et al., 2025; Tolomeo and Cascio, 2025). Moderate binding was predicted for lineage-defining transcription factors (RORC, FOXP3, TBX21, GATA3), cytokines (IL6, IFNG), and regulators of survival and signaling (BCL2, TGFBR1, HSP90AA1). While these interactions require experimental validation, they align with our functional data showing altered Th17/T_reg_ balance. Albumin (ALB) may act as a plasma carrier (Fathi et al., 2016). Predicted interactions with BCL2 and HSP90AA1 suggest effects on T cell survival, T_reg_ expansion, and antigen recognition (Liu et al., 2022b) and may contribute to the altered homeostatic balance observed. **HSP90AA1** regulates key T cell surface proteins, including those responsible for antigen recognition and costimulation (Scarneo et al., 2022).

To investigate mechanisms behind the observed immunological effects, we constructed a protein-protein interaction (PPI) network from key genes modulated in our model (Fig. 5A). Central nodes in the network, including **AHR, NQO1, KEAP1, JAK2, IL6, FOXP3, RORC** and **GATA3** suggest a convergence of oxidative stress responses and T cell mediated immune regulation. The dense interconnectivity between these hubs and peripheral genes indicates that multiple signaling pathways are co-regulated, particularly those related to **inflammation** (*IL6, JAK2*), **T cell differentiation** (*FOXP3, RORC, TBX21*), and **xenobiotic response** (*AHR, NQO1, KEAP1*). This is further supported by the results of modular analysis, where distinct clusters centered around **TGFBR1 and ALB** suggest additional axes of immune modulation presenting broader biological context (Fig. 5A). Fig. 5B presents a **network-based ranking of genes** according to their **centrality** within the immunoregulatory network. Nodes are color-coded from red to yellow, with red indicating the highest centrality scores. This visualization highlights **IL6, IFNG, CD4, JAK2** and **GATA3** as key regulators or potential bottlenecks in the system. Their central positions imply a disproportionate influence on overall network dynamics.

These findings suggest that tBHQ does not act *via* a single linear signaling cascade but rather **modulates a highly interconnected regulatory** system. We also show that **oxidative signaling** is tightly linked to these regulatory processes and together they contribute to the observed effects. These results have been illustrated with KEGG/GO analysis (Supplementary Table 1). Many of the identified signaling pathways are known to play a role in T cell development or show mechanistic overlap with T cell-mediated immune responses, autoimmune and inflammatory conditions.

## 5 Conclusion

This study addresses the immunotoxicity of tBHQ exposure and its mechanisms. It has been confirmed in numerous studies that Th17 and T_reg_ differentiation are tightly linked and that there is a functional antagonism between Th17 effectors and CD4^+^CD25^+^ T_reg_ cells. Our study provides additional evidence for this phenomenon and supports the fact that xenobiotics like tBHQ alter Th17/T_reg_ balance and T cell activation in favor of Th17 through NRF2 and possible modulation of AHR-associated pathways. These findings suggest that although tBHQ exposure may promote a regulatory phenotype under homeostatic conditions, this balance becomes destabilized during immune activation, resulting in a shift toward enhanced effector responses after subchronic tBHQ exposure. Moreover, employing integrative computational and mechanistic approaches, we also highlight the complexity of tBHQ risk assessment by predicting an array of potential targets of this chemical.

## CRediT authorship contribution statement

**Zoltán Palczert:** Investigation, Data curation. **Krisztina Németh:** Investigation, Methodology. **Nóra Fekete:** Investigation, Methodology. **Éva Pállinger:** Validation, Visualization. **Márta Békés-Kanalas:** Investigation, Methodology. **Péter Petschner:** Software, Data curation. **Edit I. Buzás:** Writing – review & editing. **Miklós Csala:** Writing – review & editing. **Viola Tamási:** Supervision, Writing – original draft.

## Funding

This work was funded by the 10.13039/501100003549Hungarian Scientific Research Fund, OTKA-PD 10829, Hungarian Brain Research Program 3.0: NAP2022-I-4/2022, János Bolyai Research Scholarship of the Hungarian Academy of Science and the New National Excellence Program of The Ministry of Human Capacities [ÚNKP-19-4-SE-09]. TKP2021-EGA-24 was implemented with the support provided by the Ministry of Innovation and Technology of Hungary from the National Research, Development and Innovation Fund, and financed under the TKP2021-EGA funding scheme. Open access funding was provided by Semmelweis University.

## Declaration of competing interest

The authors declare that they have no known competing financial interests or personal relationships that could have appeared to influence the work reported in this paper.

## Data Availability

The datasets generated and/or analyzed during the current study are available from the corresponding author on reasonable request.
