# Supplementary material for: Food preservative tBHQ modulates Th17/Treg balance through AHR/NRF2 xenosensors and increases the sensitivity of T cells to activation stimuli
Source: Curr Res Toxicol. 2026 Jul 18;11:100314. doi: 10.1016/j.crtox.2026.100314 (PMC13392857; doi:10.1016/j.crtox.2026.100314)
Supplement: Supplementary file 1 — Supplementary material [file mmc1.docx]

**Supplementary material:**

**Food preservative tBHQ modulates Th17/T_reg_ balance through AHR/NRF2 xenosensors and increases the sensitivity of T cells to activation stimuli**

Zoltán Palczert, Krisztina Németh, Nóra Fekete, Éva Pállinger, Márta Békés-Kanalas, Péter Petschner^4,^, Edit I. Buzás, Miklós Csala, Viola Tamási^, *^

**Figure S1.** Body weight changes during the treatment period. Mice were fed standard food pellets with or without 1.5% (w/w) tBHQ for 20 days. Weight is expressed as a percentage of the initial body weight on day 1. Data represent means ± SD for 6 mice per group

**Supplementary Figure 2. Representative flow cytometry plots illustrating the gating strategy used in experiments**.

**Gating of ex vivo experiments:**

**Percentage of CD3^+^CD4^+^ cells after autoMACS separation.** CD4+ cells were separated from splenocytes by positive selection using an autoMACS system. The CD3/CD4 positivity of the cells was assessed with FACS analisys. As shown on the dot plot, experiments were performed only with cell preparations in which the portion of CD3^+^CD4^+^ cells exceeded 85%.

**
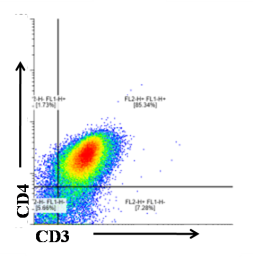
**

**Representative gating strategy of effector T cells from separated, CD4^+^ cells.** The cells were gated based on FSC/SSC and than, different T cells were characterized using specific marker protein (INFγ for Th1 cells; IL-4 for Th2 cells; IL-17 for Th17 cells. Cells in upper right quartile were concidered as positive cells for the specific marker.

**Representative gating strategy for Treg cells separated from CD4^+^ cells.** First, cells were gated based on FSC and SSC parameters. Subsequently, Treg specific markers, CD25 and FOXP3 were analysed. CD25^+^FOXP3^+^ cells (upper right quartile) were further subdivided into two populations: CD25^high^ (red square) and CD25^low^.

**Gating of in vitro experiments:**

**Gating strategy for IL-17–producing CD4⁺ T cells according to FlowCellect^TM^ Mouse Th17 Differentiation Tool Kit.** From live cells were selected with eFluor and lymphocytes were gated based on FSC/SSC according to size and granularity. CD4⁺ T cells were then identified within the lymphocyte gate, and IL-17 expression was analyzed within the CD4⁺ population to identify Th17 cells.

**Gating strategy for the identification of regulatory T cells based on FlowCellect^TM^ Mouse Viable T_reg_ characterization kit.** Lymphocytes were first gated based on forward scatter (FSC) and side scatter (SSC). Then, CD4⁺ cells were gated from the lymphocytes. It was followed by gating on CD25 expression within the CD4⁺ population. Finally, FoxP3 expression was analyzed within the CD25⁺ CD4⁺ gate to identify Tregs.

**Table 1. GO and KEGG functional annotation clustering by DAVID Bioinformatics. Classification Stringency: Medium**

| **Name** | **p-value** | **Genes** |
| --- | --- | --- |
| hsa04659:Th17 cell differentiation | 2.591447954207353E-13 | IL6, IFNG, TBX21, RORC, GATA3, AHR, JAK2, FOXP3, TGFBR1 |
| hsa05321:Inflammatory bowel disease | 1.80165335165096E-8 | IL6, IFNG, TBX21, RORC, GATA3, FOXP3 |
| GO:0045893~positive regulation of DNA-templated transcription | 1.8172373405621174E-6 | IL6, TBX21, RORC, GATA3, AHR, FOXP3, TGFBR1 |
| GO:0045944~positive regulation of transcription by RNA polymerase II | 3.7014075449218327E-5 | IL6, IFNG, TBX21, GATA3, AHR, JAK2, FOXP3 |
| GO:0009615~response to virus | 3.920705385181122E-5 | IFNG, TBX21, GATA3, FOXP3 |
| GO:0032703~negative regulation of interleukin-2 production | 1.6019496147749157E-4 | TBX21, GATA3, FOXP3 |
| GO:0000122~negative regulation of transcription by RNA polymerase II | 1.6880780326518835E-4 | IFNG, TBX21, RORC, KEAP1, GATA3, FOXP3 |
| hsa04658:Th1 and Th2 cell differentiation | 2.567054580001993E-4 | IFNG, TBX21, GATA3, JAK2 |
| GO:0045892~negative regulation of DNA-templated transcription | 3.038978743244564E-4 | IFNG, TBX21, GATA3, AHR, FOXP3 |
| GO:1990837~sequence-specific double-stranded DNA binding | 3.0719663291672844E-4 | TBX21, RORC, GATA3, AHR, FOXP3 |
| GO:0003700~DNA-binding transcription factor activity | 4.0317157635961305E-4 | TBX21, RORC, GATA3, AHR, FOXP3 |
| GO:0001227~DNA-binding transcription repressor activity, RNA polymerase II-specific | 5.582477471730564E-4 | TBX21, RORC, GATA3, FOXP3 |
| GO:0043565~sequence-specific DNA binding | 0.0010759502164587452 | TBX21, RORC, BCL2, FOXP3 |
| GO:0000785~chromatin | 0.0030496260862183577 | TBX21, RORC, GATA3, AHR, FOXP3 |
| GO:0000981~DNA-binding transcription factor activity, RNA polymerase II-specific | 0.0058745294141108505 | TBX21, RORC, GATA3, AHR, FOXP3 |
| GO:0000976~transcription cis-regulatory region binding | 0.00948130449148941 | TBX21, GATA3, AHR |
| GO:0003677~DNA binding | 0.010929592043963559 | ALB, RORC, GATA3, AHR, FOXP3 |
| GO:0000978~RNA polymerase II cis-regulatory region sequence-specific DNA binding | 0.03301300056340416 | TBX21, RORC, GATA3, FOXP3 |
| GO:0001228~DNA-binding transcription activator activity, RNA polymerase II-specific | 0.03698729307892917 | TBX21, GATA3, FOXP3 |
| GO:0006357~regulation of transcription by RNA polymerase II | 0.08169614450664692 | TBX21, RORC, AHR, FOXP3 |
| GO:0008270~zinc ion binding | 0.40291626453935153 | RORC, GATA3, FOXP3 |
| GO:0048538~thymus development | 4.026496576169867E-4 | BCL2, GATA3, TGFBR1 |
| GO:0009791~post-embryonic development | 9.668329974974611E-4 | BCL2, GATA3, TGFBR1 |
| GO:0008584~male gonad development | 0.001974341632856531 | BCL2, GATA3, TGFBR1 |
| GO:0006959~humoral immune response | 6.948333977108423E-6 | IL6, IFNG, BCL2, GATA3 |
| GO:0008284~positive regulation of cell population proliferation | 1.9746431222178063E-4 | IL6, IFNG, BCL2, JAK2, TGFBR1 |
| GO:0007259~cell surface receptor signaling pathway via JAK-STAT | 5.642284829141211E-4 | IL6, IFNG, JAK2 |
| GO:0032731~positive regulation of interleukin-1 beta production | 7.301637835735119E-4 | IL6, IFNG, JAK2 |
| hsa04630:JAK-STAT signaling pathway | 0.001446584353117216 | IL6, IFNG, BCL2, JAK2 |
| GO:0098586~cellular response to virus | 0.0015363808685609698 | IL6, IFNG, JAK2 |
| hsa05152:Tuberculosis | 0.0018213232533246541 | IL6, IFNG, BCL2, JAK2 |
| hsa05168:Herpes simplex virus 1 infection | 0.0018213232533246541 | IL6, IFNG, BCL2, JAK2 |
| GO:0032760~positive regulation of tumor necrosis factor production | 0.002082894259913002 | IL6, IFNG, JAK2 |
| GO:0051607~defense response to virus | 0.00927237131164091 | IL6, IFNG, BCL2 |
| hsa04066:HIF-1 signaling pathway | 0.009987740679385942 | IL6, IFNG, BCL2 |
| hsa05164:Influenza A | 0.023597901158763306 | IL6, IFNG, JAK2 |
| GO:0006955~immune response | 0.04170528106580895 | IL6, IFNG, JAK2 |
| hsa05200:Pathways in cancer | 3.856550278658965E-5 | NQO1, IL6, IFNG, BCL2, KEAP1, JAK2, TGFBR1 |
| GO:0008284~positive regulation of cell population proliferation | 1.9746431222178063E-4 | IL6, IFNG, BCL2, JAK2, TGFBR1 |
| hsa04933:AGE-RAGE signaling pathway in diabetic complications | 3.2758125787533065E-4 | IL6, BCL2, JAK2, TGFBR1 |
| GO:0006915~apoptotic process | 5.084452414221426E-4 | IFNG, BCL2, AHR, JAK2, TGFBR1 |
| hsa05161:Hepatitis B | 0.0013257702800118375 | IL6, BCL2, JAK2, TGFBR1 |
| hsa04630:JAK-STAT signaling pathway | 0.001446584353117216 | IL6, IFNG, BCL2, JAK2 |
| hsa05152:Tuberculosis | 0.0018213232533246541 | IL6, IFNG, BCL2, JAK2 |
| hsa05168:Herpes simplex virus 1 infection | 0.0018213232533246541 | IL6, IFNG, BCL2, JAK2 |
| GO:0043066~negative regulation of apoptotic process | 0.0033831284552051266 | NQO1, IL6, BCL2, TGFBR1 |
| hsa01521:EGFR tyrosine kinase inhibitor resistance | 0.005389375385686642 | IL6, BCL2, JAK2 |
| hsa05145:Toxoplasmosis | 0.010339773089763074 | IFNG, BCL2, JAK2 |
| GO:0043065~positive regulation of apoptotic process | 0.016690457446574086 | IL6, BCL2, TGFBR1 |
| hsa04217:Necroptosis | 0.020141669794467663 | IFNG, BCL2, JAK2 |
| hsa05417:Lipid and atherosclerosis | 0.03561493981688631 | IL6, BCL2, JAK2 |
| GO:0030154~cell differentiation | 0.06927294492291611 | IL6, JAK2, TGFBR1 |
| hsa04151:PI3K-Akt signaling pathway | 0.08945789976083944 | IL6, BCL2, JAK2 |
| GO:0016020~membrane | 0.640907873519333 | NQO1, BCL2, JAK2, TGFBR1 |
| GO:0008284~positive regulation of cell population proliferation | 1.9746431222178063E-4 | IL6, IFNG, BCL2, JAK2, TGFBR1 |
| hsa05142:Chagas disease | 0.008799517540153904 | IL6, IFNG, TGFBR1 |
| GO:0010628~positive regulation of gene expression | 0.035147506235688686 | IL6, IFNG, TGFBR1 |
| hsa04060:Cytokine-cytokine receptor interaction | 0.06367707292328256 | IL6, IFNG, TGFBR1 |
| GO:0008285~negative regulation of cell population proliferation | 0.0020669258841609123 | IL6, GATA3, JAK2, FOXP3 |
| GO:0006366~transcription by RNA polymerase II | 0.009420786724074077 | GATA3, JAK2, FOXP3 |
| GO:0006338~chromatin remodeling | 0.06471477923063462 | GATA3, JAK2, FOXP3 |

Table 2: **G:Profiler enrichment analysis. Term size was set to 50-65.**

| **Source** | **Term name** | **Adjusted p-value** | **Intersections** |
| --- | --- | --- | --- |
| GO:MF | E-box binding | 0.008229970332891464 | ENSG00000106546,ENSG00000107485 |
| GO:MF | ligand-modulated transcription factor activity | 0.008229970332891464 | ENSG00000106546,ENSG00000143365 |
| GO:MF | nuclear receptor activity | 0.008229970332891464 | ENSG00000106546,ENSG00000143365 |
| GO:BP | T-helper 17 type immune response | 3.674199539143191e-8 | ENSG00000136244,ENSG00000143365,ENSG00000073861,ENSG00000049768,ENSG00000096968 |
| GO:BP | T cell selection | 5.92202682578654e-8 | ENSG00000171791,ENSG00000136244,ENSG00000073861,ENSG00000107485,ENSG00000049768 |
| GO:BP | positive T cell selection | 0.0000010230168112195934 | ENSG00000171791,ENSG00000136244,ENSG00000073861,ENSG00000049768 |
| GO:BP | regulation of CD4-positive, alpha-beta T cell differentiation | 0.00000197335987080789 | ENSG00000111537,ENSG00000073861,ENSG00000107485,ENSG00000049768 |
| GO:BP | thymus development | 0.00005362648639159176 | ENSG00000171791,ENSG00000107485,ENSG00000106799 |
| GO:BP | leukocyte activation involved in inflammatory response | 0.00005665119332230849 | ENSG00000111537,ENSG00000136244,ENSG00000096968 |
| GO:BP | regulation of tyrosine phosphorylation of STAT protein | 0.00007143973656984596 | ENSG00000111537,ENSG00000136244,ENSG00000096968 |
| GO:BP | glial cell activation | 0.00007351318313642502 | ENSG00000111537,ENSG00000136244,ENSG00000096968 |
| GO:BP | negative regulation of lymphocyte mediated immunity | 0.0000741570110249853 | ENSG00000106546,ENSG00000073861,ENSG00000049768 |
| GO:BP | regulation of epithelial cell apoptotic process | 0.0000741570110249853 | ENSG00000171791,ENSG00000136244,ENSG00000096968 |
| GO:BP | regulation of nitric oxide biosynthetic process | 0.0000741570110249853 | ENSG00000111537,ENSG00000096968,ENSG00000080824 |
| GO:BP | regulation of nitric oxide metabolic process | 0.0000795708631474488 | ENSG00000111537,ENSG00000096968,ENSG00000080824 |
| GO:BP | positive regulation of interleukin-1 beta production | 0.0000795708631474488 | ENSG00000111537,ENSG00000136244,ENSG00000096968 |
| GO:BP | tyrosine phosphorylation of STAT protein | 0.0000795708631474488 | ENSG00000111537,ENSG00000136244,ENSG00000096968 |
| GO:BP | regulation of protein import into nucleus | 0.00008289324603206673 | ENSG00000111537,ENSG00000096968,ENSG00000080824 |
| GO:BP | negative regulation of adaptive immune response  based on somatic recombination of immune receptors  built from immunoglobulin superfamily domains | 0.00008551905795297073 | ENSG00000106546,ENSG00000073861,ENSG00000049768 |
| GO:BP | interleukin-2 production | 0.0000926984740043418 | ENSG00000073861,ENSG00000107485,ENSG00000049768 |
| GO:BP | regulation of interleukin-2 production | 0.0000926984740043418 | ENSG00000073861,ENSG00000107485,ENSG00000049768 |
| GO:BP | positive regulation of nucleocytoplasmic transport | 0.00009607987903109851 | ENSG00000111537,ENSG00000096968,ENSG00000080824 |
| GO:BP | axon regeneration | 0.0022385609167342407 | ENSG00000171791,ENSG00000096968 |
| GO:BP | negative regulation of alpha-beta T cell activation | 0.0022385609167342407 | ENSG00000073861,ENSG00000049768 |
| GO:BP | negative regulation of T cell differentiation | 0.002387044931570549 | ENSG00000073861,ENSG00000049768 |
| GO:BP | regulation of platelet activation | 0.00246477315793601 | ENSG00000136244,ENSG00000096968 |
| GO:BP | response to amine | 0.00246477315793601 | ENSG00000181019,ENSG00000096968 |
| GO:BP | negative regulation of leukocyte apoptotic process | 0.0025331653792975643 | ENSG00000171791,ENSG00000143365 |
| GO:BP | positive regulation of alpha-beta T cell differentiation | 0.0026121772834014814 | ENSG00000111537,ENSG00000049768 |
| GO:BP | positive regulation of immunoglobulin production | 0.0026121772834014814 | ENSG00000136244,ENSG00000073861 |
| GO:BP | positive regulation of epithelial to mesenchymal transition | 0.0026797739856613493 | ENSG00000136244,ENSG00000106799 |
| GO:BP | somatic recombination of immunoglobulin genes involved in immune response | 0.0026797739856613493 | ENSG00000073861,ENSG00000049768 |
| GO:BP | somatic diversification of immunoglobulins involved in immune response | 0.0026797739856613493 | ENSG00000073861,ENSG00000049768 |
| GO:BP | isotype switching | 0.0026797739856613493 | ENSG00000073861,ENSG00000049768 |
| GO:BP | neuron projection regeneration | 0.0027215120807974956 | ENSG00000171791,ENSG00000096968 |
| GO:BP | immunoglobulin production involved in immunoglobulin-mediated immune response | 0.0028746618394959278 | ENSG00000073861,ENSG00000049768 |
| GO:BP | regulation of interleukin-10 production | 0.0028746618394959278 | ENSG00000136244,ENSG00000049768 |
| GO:BP | regulation of lymphocyte apoptotic process | 0.0028746618394959278 | ENSG00000171791,ENSG00000143365 |
| GO:BP | interleukin-10 production | 0.0028746618394959278 | ENSG00000136244,ENSG00000049768 |
| GO:BP | intrinsic apoptotic signaling pathway in response to oxidative stress | 0.0028746618394959278 | ENSG00000171791,ENSG00000096968 |
| GO:BP | T cell apoptotic process | 0.0029547725291280877 | ENSG00000171791,ENSG00000143365 |
| GO:BP | negative regulation of lymphocyte differentiation | 0.0029547725291280877 | ENSG00000073861,ENSG00000049768 |
| GO:BP | regulation of extracellular matrix organization | 0.0031338804693709084 | ENSG00000136244,ENSG00000106799 |
| GO:BP | regulation of immunoglobulin mediated immune response | 0.0032105207654914217 | ENSG00000073861,ENSG00000049768 |
| GO:BP | regulation of B cell mediated immunity | 0.0032105207654914217 | ENSG00000073861,ENSG00000049768 |
| GO:BP | regulation of SMAD protein signal transduction | 0.0032105207654914217 | ENSG00000096968,ENSG00000106799 |
| GO:BP | somatic recombination of immunoglobulin gene segments | 0.0032105207654914217 | ENSG00000073861,ENSG00000049768 |
| GO:BP | macrophage differentiation | 0.003293444092127621 | ENSG00000111537,ENSG00000107485 |
| GO:BP | response to ischemia | 0.003293444092127621 | ENSG00000171791,ENSG00000181019 |
| GO:BP | regulation of filopodium assembly | 0.04907730714867427 | ENSG00000106799 |
| GO:BP | coronary vasculature development | 0.04907730714867427 | ENSG00000106799 |
| GO:BP | cardiac muscle cell proliferation | 0.04907730714867427 | ENSG00000106799 |
| GO:BP | epithelial cell differentiation involved in kidney development | 0.04907730714867427 | ENSG00000107485 |
| GO:BP | monocyte chemotaxis | 0.04907730714867427 | ENSG00000136244 |
| GO:BP | activin receptor signaling pathway | 0.04907730714867427 | ENSG00000106799 |
| GO:BP | postsynaptic signal transduction | 0.04907730714867427 | ENSG00000096968 |
| GO:BP | fear response | 0.04907730714867427 | ENSG00000171791 |
| GO:BP | protein deacylation | 0.04907730714867427 | ENSG00000111537 |
| GO:BP | negative regulation of DNA recombination | 0.04977329168403915 | ENSG00000049768 |
| GO:BP | positive regulation of DNA biosynthetic process | 0.04977329168403915 | ENSG00000080824 |
| GO:BP | regulation of lamellipodium organization | 0.04977329168403915 | ENSG00000080824 |
| GO:BP | regulation of lipid storage | 0.04977329168403915 | ENSG00000136244 |
| GO:BP | cellular response to cAMP | 0.04977329168403915 | ENSG00000106546 |
| KEGG | Inflammatory bowel disease | 2.0301979452167325e-8 | ENSG00000111537,ENSG00000136244,ENSG00000143365,ENSG00000073861,ENSG00000107485,ENSG00000049768 |
